# Alpha6beta4 integrin crosslinking induces EGFR clustering and promotes EGF-mediated Rho activation in breast cancer

**DOI:** 10.1186/1756-9966-28-67

**Published:** 2009-05-26

**Authors:** Michael Z Gilcrease, Xiao Zhou, Xiaolin Lu, Wendy A Woodward, Brian E Hall, Phillip J Morrissey

**Affiliations:** 1 Department of Pathology, The University of Texas M.D. Anderson Cancer Center, 1515 Holcombe Blvd, Houston, TX, USA; 2 Department of Radiation Oncology, The University of Texas M.D. Anderson Cancer Center, 1515 Holcombe Blvd, Houston, TX, USA; 3 Amnis Corporation, 2505 Third Avenue, Suite 210, Seattle, WA, USA

## Abstract

**Background:**

The α6β4 integrin is overexpressed in the basal subtype of breast cancer and plays an important role in tumor cell motility and invasion. EGFR is also overexpressed in the basal subtype of breast cancer, and crosstalk between α6β4 integrin and EGFR appears to be important in tumor progression.

**Methods:**

We evaluated the effects of α6β4 crosslinking on the distribution and function of EGFR in breast carcinoma cell line MDA-MB-231. Receptor distribution was evaluated by fluorescence microscopy and multispectral imaging flow cytometry, and ligand-mediated EGFR signaling was evaluated using Western blots and a Rho pull-down assay.

**Results:**

Antibody-mediated crosslinking of α6β4 integrin was sufficient to induce cell-surface clustering of not only α6β4 but also EGFR in nonadherent cells. The induced clustering of EGFR was observed minimally after 5 min of integrin crosslinking but was more prominent after 15 min. EGFR clustering had minimal effect on the phosphorylation of Akt or Erk1,2 in response to EGF in suspended cells or in response to HB-EGF in adherent cells. However, EGFR clustering induced by crosslinking α6β4 had a marked effect on Rho activation in response to EGF.

**Conclusion:**

Crosslinking α6β4 integrin in breast carcinoma cells induces EGFR clustering and preferentially promotes Rho activation in response to EGF. We hypothesize that this integrin-EGFR crosstalk may facilitate tumor cell cytoskeletal rearrangements important for tumor progression.

## Background

Integrins are an important class of cell surface receptors that recognize extracellular matrix proteins and allow the cell's microenvironment to help regulate intracellular signaling events[1,2]. Binding to multivalent ligands results in integrin crosslinking, which activates a signaling process that induces integrin clustering within the plasma membrane[3,4]. Clustering of integrins in vitro can also be investigated with crosslinking antibodies, which provide greater specificity than most integrin ligands[5]. In the process of integrin clustering, integrins that are diffusely distributed throughout the membrane dissociate from their cytoskeletal contacts and aggregate in particular regions of the membrane, where they form large complexes with new attachments to the cytoskeleton[6,7]. In addition to activating the individual integrin heterodimers, the clustering of integrins leads to recruitment of other signaling molecules to the plasma membrane [1-4].

Activated integrins are known to regulate growth factor receptor signaling in normal and malignant cells[8,9]. Integrin-growth factor receptor crosstalk is important for many growth factor receptor-mediated functions, including cell proliferation, survival, motility and invasion[8,9]. The α6β4 integrin, a receptor for most laminins that is normally expressed in the myoepithelial cell layer of benign breast epithelium[10], is upregulated in the aggressive basal subtype of invasive breast cancer[11]. EGFR is also overexpressed in this subgroup of breast cancers[11], and in-vitro data suggest that crosstalk between α6β4 integrin and EGFR may be important in the progression of this basal subtype of breast cancers [12-14].

EGFR converts from an inactive monomeric form to an active homodimer upon stimulation by its ligand[15,16], and cell surface clusters of activated EGFR homodimers are known to occur [17-19]. We showed previously that α6β4 integrin crosslinking induces PI3K-dependent cell surface clustering of α6β4 integrin in breast carcinoma cells[20]. Because integrin clusters are known to recruit other molecules to membrane complexes, we hypothesized that α6β4 clustering might lead to the redistribution and clustering of EGFR on the tumor cell surface. Moreover, because cell surface clustering of a variety of receptors, including EGFR, has been shown to augment receptor function[5,17-19], we hypothesized that α6β4 integrin-induced EGFR clustering might augment particular tumor cell responses to EGF. This might be one mechanism whereby integrins allow the microenvironment to regulate tumor cell behavior.

Here we report the effects of adhesion-independent α6β4 integrin crosslinking on the distribution and function of EGFR in MDA-MB-231 breast carcinoma cells, known to express high levels of α6β4 integrin and EGFR typical of basal-like breast carcinomas.

## Methods

### Cell Culture

Breast carcinoma cell line MDA-MB-231, an aggressive breast carcinoma cell line derived from the pleural effusion of a patient with metastatic carcinoma, was cultured in Eagle's Minimum Essential Medium (MEM) supplemented with 5% fetal bovine serum (FBS), L-glutamine, sodium pyruvate, and nonessential amino acids and vitamins (Gibco). The cells were maintained in monolayer culture in a humidified incubator at 37°C in an atmosphere of 5% CO_2 _and 95% air.

### Receptor Clustering and Fluorescence Microscopy

Cells were serum-starved overnight, trypsinized from the culture dishes and washed twice with PBS. The cells were then resuspended in MEM containing 0.1% bovine serum albumin at a concentration of 5 × 10^6 ^cells/ml. For integrin crosslinking, cells in suspension were incubated with mouse monoclonal anti-β4 (clone 3E1, Chemicon) on ice for 30 min, washed, and then incubated with either rabbit anti-mouse IgG (Sigma) or rabbit IgG control at 37°C for various time periods. Following fixation in 2% paraformaldehyde, immunofluorescence staining for α6β4 was performed using mouse monoclonal anti-β4 (clone ELF1, Novocastra) as the primary antibody and FITC-labeled anti-mouse IgG (Zymed) as the secondary. Staining for EGFR was performed using FITC-rat anti-EGFR (clone ICR10, Serotec). The labeled cells were cytocentrifuged onto a glass slide and evaluated by fluorescence microscopy.

### Multispectral Imaging Flow Cytometry

MDA-MB-231 cells were treated as above, stained with FITC-rat anti-EGFR on ice, fixed in paraformaldehyde, and then permeabilized and stained with DRAQ5 to 10 μM (Biostatus, Shepshed, United Kingdom). Induced clustering of EGFR was analyzed by multispectral imaging analysis of cells in flow using the ImageStream™ (Amnis Corporation, Seattle, Washington). Briefly, this system illuminates hydrodynamically focused cells with a 488 nm laser oriented perpendicular to the collection axis and simultaneously transilluminates along the collection axis by a brightfield light source. The light is collected with an imaging objective lens and projected on a CCD operating in time-delay integration (TDI) mode. Prior to projection on the CCD, the light is passed through a multispectral optical system that decomposes and redirects the light into multiple channels, each corresponding to a different spectral band. The images are spatially offset from each other to facilitate image processing and quantitation. For this study, a channel for a brightfield image, a 500–560 nm channel for FITC, and a 660–735 nm channel for DRAQ5 were used.

Following spectral compensation, calculation of fluorescence intensity parameters was performed using the Image Data Exploration and Analysis Software (IDEAS) package (Amnis Corporation). EGFR clustering was quantified using a "small spot total" classifier that measures small regions of continuously connected bright intensity over a 7-pixel octagonal area, normalized to mean intensity. The normalized value is expressed as "Bright Detail Intensity-FITC". Bivariate dot plots of "Bright Detail Intensity-FITC" on the Y axis and "Area Threshold 30%" on the X axis were produced. "Area Threshold 30%" is the area of the pixels in the brightest 30^th ^percentile within the image. As EGFR condenses into a small number of brighter pixels, the Area Threshold 30% decreases. Conversely, when EGFR is uniformly distributed over a large number of pixels, the brightest 30% of the pixels is much closer to the mean pixel value, and the area is much larger. Values along the Y axis measure the degree of punctate staining, and values along the X axis measure diffuseness of staining. Dots to the left of an arbitrary diagonal (representing cells with clustered EGFR) were quantified before and after crosslinking cell surface α6β4 integrin.

### Western Blotting

After cross-linking α6β4 on cells in suspension, cells were exposed to EGF (10 ng/ml) or buffer alone at 37°C for various time periods, then lysed on ice for 30 min with lysis buffer containing 50 mM HEPES at pH 7.4, 150 mM NaCl, 1% Triton X-100, 1 mM CaCl_2_, 1 mM MgCl_2_, 10% glycerol, 100 mM NaF, 1 mM sodium orthovanadate, 10 mM sodium pyrophosphate, 1 mM PMSF, 10 μg/ml leupeptin, and 10 μg/ml aprotinin. Aliquots of lysates with equal amounts of total protein were separated on 7.5% SDS-PAGE gels under reducing conditions and transferred to nitrocellulose filters. Filters were probed with rabbit polyclonal antibodies to phospho-Akt (Ser473) (Cell Signaling) and phospho-Erk1,2 (Thr202/Tyr204) (Cell Signaling), and membranes were subsequently stripped and probed for total Akt and total Erk1,2. Alternatively, cells were treated with anti-β4 on ice for 40 min and applied to plates coated with anti-mouse IgG + heparin-binding EGF-like growth factor (HB-EGF) or rabbit IgG control + HB-EGF for up to 1 hr, and Western blots were similarly probed. After incubating the filters with horseradish peroxidase-linked streptavidin (Vector), proteins were detected with the ECL Western Blotting Detection Reagents (Amersham) for various time periods.

### Rho Pull-down Assay

To determine whether integrin-induced EGFR clustering augments Rho activation in response to EGF, α6β4 was crosslinked on cells in suspension, and the cells were treated with EGF (10 ng/ml) or buffer alone for 15 min or 30 min. A Rho pull-down assay with GST-tagged Rho-binding domain of Rhotekin on glutathione-agarose beads was performed (Upstate Cell Signaling Solutions, Temecula, CA), and a Western blot was probed with anti-Rho. MDA-MB-231 cell extract loaded with 100 μM GTPγS for 30 min at 30°C was used as a positive control, and the same extract loaded with 1 mM GDP was used as a negative control.

## Results

**Figure 1:**
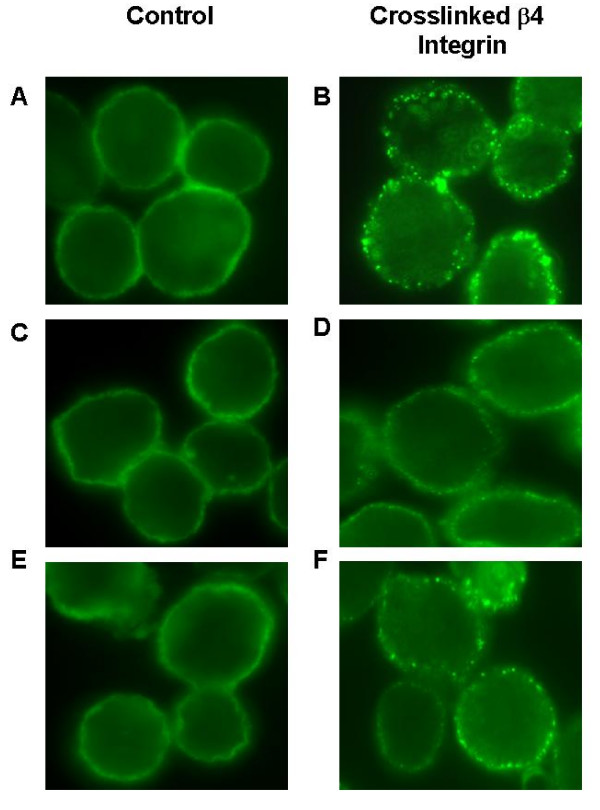
**Induced clustering of α6β4 (B) and EGFR (D, F)**. MDA-MB-231 cells were exposed to anti-β4 on ice, followed by control rabbit IgG (A, C, E) or rabbit anti-mouse IgG (B, D, F) at 37°C to crosslink α6β4 for 30 min (A, B), 5 min (C, D), or 15 min (E, F). Cells were stained with either FITC-labeled anti-mouse IgG to detect β4 (A, B) or FITC-labeled anti-EGFR (C-F).

The effect of α6β4 integrin crosslinking on cell surface EGFR distribution in MDA-MB-231 breast carcinoma cells was assessed by immunofluorescence microscopy after incubating the cells first with mouse monoclonal anti-β4 on ice, followed by either rabbit IgG control or rabbit anti-mouse IgG at 37°C to crosslink α6β4. Crosslinking the integrin on nonadherent cells was sufficient to induce cell-surface clustering of not only α6β4 (Figure 1A and 1B) but also EGFR. Integrin-induced EGFR clustering was observed minimally after 5 min of integrin crosslinking (Figure 1C and 1D), and the extent of EGFR clustering increased at 15 min (Figure 1E and 1F).

**Figure 2:**
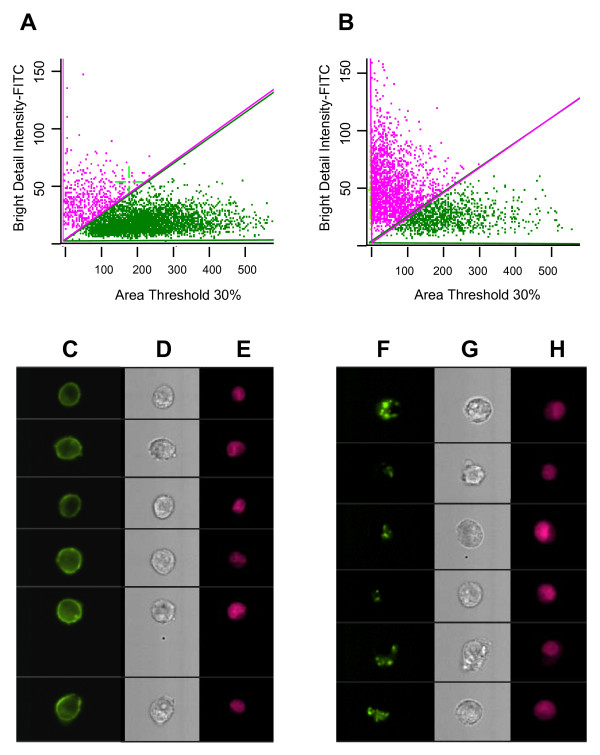
**Bivariate dot plots of "Area Threshold 30%" representing diffuseness of staining on the X axis and "Bright Detail Intensity-FITC" representing the degree of punctuate staining on the Y axis (see Materials and Methods)**. MDA-MB-231 cells were exposed to anti-β4 on ice, followed by control rabbit IgG (A) or rabbit anti-mouse IgG (B) at 37°C to crosslink α6β4 for 30 min. Cells were stained with FITC-labeled anti-EGFR and nuclear stain DRAQ5 and run on the ImageStream™. Representative brightfield (BF) and fluorescent images from gated cells to the right of the diagonal in B show a diffuse cell surface distribution of EGFR (C-E), whereas representative images of gated cells to the left of the diagonal in B show a clustered distribution of EGFR (F-H).

Induced EGFR clustering was quantified by multispectral imaging flow cytometry using the ImageStream™. Incubation with integrin crosslinking antibodies or control antibodies was performed as before, and cells were stained with FITC-rat anti-EGFR on ice and fixed in paraformaldehyde. Cells were then permeabilized, stained with the nuclear stain DRAQ5, and run on the ImageStream™. Using the ImageStream's IDEAS software, bivariate dot plots of "Area Threshold 30%" on the X axis and "Bright Detail Intensity-FITC" representing the degree of punctuate staining on the Y axis were produced (see Materials and Methods). Whereas only 10% of the baseline tumor cell population fell within the region on the bivariate dot plot to the left of the diagonal, representing cells with clustered EGFR above an arbitrarily defined threshold (Figure 2A), the proportion increased to 65% after crosslinking α6β4 integrin (Figure 2B). Representative images from gated cells to the right of the diagonal show a diffuse cell surface distribution of EGFR (Figure 2C–E), whereas representative images of gated cells to the left of the diagonal show a clustered distribution of EGFR (Figure 2F–H).

**Figure 3:**
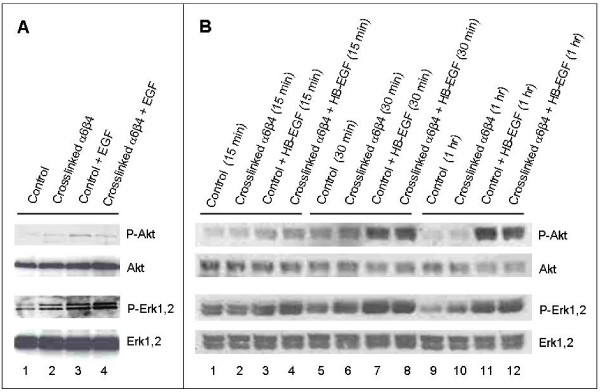
**The effect of α6β4 crosslinking on EGFR signaling following treatment with EGF (A) or HB-EGF (B)**. A) MDA-MB-231 cells in suspension were exposed to anti-β4 on ice, followed by control rabbit IgG (lanes 1 and 3) or rabbit anti-mouse IgG (lanes 2 and 4) at 37°C for 30 min to crosslink α6β4, with (lanes 3 and 4) or without (lanes 1 and 2) subsequent addition of EGF (10 ng/ml) for 5 min. B) MDA-MB-231 cells were exposed to anti-β4 on ice, then added to plates coated with control rabbit IgG (lanes 1, 3, 5, 7, 9 and 11) or rabbit anti-mouse IgG (lanes 2, 4, 6, 8, 10, or 12) at 37°C to crosslink α6β4, in the presence (lanes 3, 4, 7, 8, 11, and 12) or absence(lanes 1, 2, 5, 6, 9, and 10) of simultaneous coating with HB-EGF. Western blots prepared from cell lysates were probed for phospho-Akt and phospho-Erk1,2, then stripped and probed for total Akt and total Erk1,2.

To determine whether integrin-induced clustering of EGFR affects tumor cell response to EGF, MDA-MB-231 cells were exposed to mouse monoclonal anti-β4 on ice, followed by control rabbit IgG or rabbit anti-mouse IgG to induce integrin and EGFR clustering, in the presence or absence of EGF (10 ng/ml). Western blots were prepared from cell lysates and probed for phospho-Akt and phospho-Erk1,2, then stripped, and probed again for total Akt and total Erk1,2 (Figure 3A). In suspended cells, there was only a very minimal, if any, effect of EGFR clustering on EGF-stimulated Akt and Erk1,2 phosphorylation. Crosslinking α6β4 by itself resulted in only a very small to equivocal increase in phospho-Akt (*lane 2*). EGF in the absence of α6β4 crosslinking did stimulate Akt phosphorylation (*lane 3*), but the effect appeared to be abrogated in the presence of α6β4 crosslinking (*lane 4*). Crosslinking α6β4 produced a small increase in phospho-Erk1,2 (*lane 2*), as did the addition of EGF (*lane 3*), but the two together did not clearly have more than an additive effect (*lane 4*).

Alternatively, to evaluate effects on adherent cells, the cells were exposed to mouse monoclonal anti-β4 in suspension on ice, then added to plates coated with control rabbit IgG or rabbit anti-mouse IgG to crosslink α6β4, with or without a substrate of HB-EGF (Figure 3B). Crosslinking α6β4 in adherent cells in the absence of HB-EGF produced a slight increase in phosphorylation of Erk1,2 at 1 hr (*lane 10*). However, crosslinking the integrin in adherent cells did not appear to enhance phosphorylation of either Akt or Erk1,2 in response to HB-EGF.

**Figure 4:**
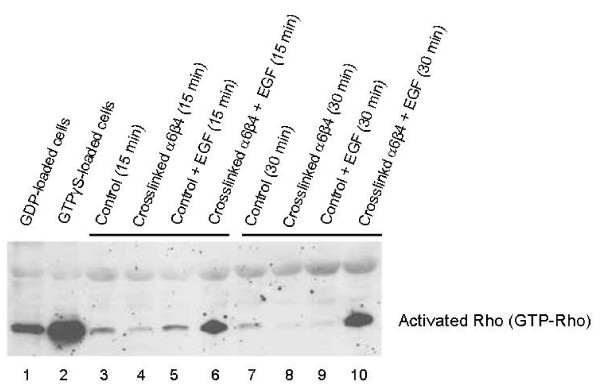
**The effect of α6β4 crosslinking on EGF-mediated Rho activation**. MDA-MB-231 cells were incubated with anti-β4 on ice, followed by control rabbit IgG (lanes 3, 5, 7 and 9) or rabbit anti-mouse IgG (lanes 4, 6, 8, and 10) at 37°C to crosslink α6β4 for 15 min (lanes 3–6) or 30 min (lanes 7–10) in the presence (lanes 5, 6, 9, and 10) or absence (lanes 3, 4, 7, and 8) of EGF (10 ng/ml). Rho activation was assayed using a Rho pull-down assay with GST-tagged Rhotekin Rho-binding domain on glutathione-agarose beads. Negative and positive controls were MDA-MB-231 cell extracts loaded for 30 min at 30°C with 1 mM GDP (lane 1) or 100 μM GTPγS (lane 2), respectively.

In contrast, crosslinking α6β4 integrin on cells in suspension to induce cell surface clustering of EGFR had a marked effect on Rho activation in response to EGF (Figure 4). EGF in the absence of α6β4 crosslinking did not induce Rho activation in suspended MDA-MB-231 cells at 15 and 30 min (*lanes 5 *and *9*), and crosslinking α6β4 in the absence of EGF even produced a slight decrease in activated Rho after 15 min and 30 min of integrin crosslinking (*lanes 4 *and *8*). However, crosslinking α6β4 in the presence of EGF produced a marked effect on Rho activation after 15 and 30 min (*lanes 6 *and *10*).

## Discussion

We observed that crosslinking α6β4 integrin in breast carcinoma cells in suspension induced cell surface clustering of EGFR. Under these conditions, although no significant change in EGF-stimulated signaling to Akt or Erk1,2 was observed, a marked increase in Rho activation occurred in response to EGF. The association between α6β4-induced EGFR clustering and a selective increase in EGFR signaling to Rho in response to EGF in nonadherent tumor cells suggests that in certain conditions, α6β4 integrin regulation of EGFR can selectively augment some aspects of EGFR signaling without stimulating others. We hypothesize that tumor cells in nonadherent or less adherent conditions, such as circulating or migrating tumor cells, might selectively regulate EGFR to enhance chemotaxis or motility at the expense of growth and survival signaling.

As adhesion receptors for extracellular matrix and regulators of intracellular signaling, integrins provide an important link between the cell and its microenvironment [1-3]. By modulating intracellular signaling pathways, integrins help to maintain cellular functions appropriate for the cell's particular location. The α6β4 integrin is a receptor for most laminins, including laminin-5, a component of the epithelial cell basement membrane[21]. It is normally expressed in the basal aspect of epithelial cells, where it functions as a component of hemidesmosomes[21,22]. In breast epithelium, α6β4 is principally expressed in the myoepithelium, which comprises the outer cell layer in contact with surrounding stroma[10].

Although generally quiescent, myoepithelial cells are known to proliferative and move through the adjacent stroma in some physiologic conditions[23]. Breast cancers that overexpress α6β4 may similarly have an increased capacity for stromal invasion. A role for α6β4 in tumor cell invasion is supported by in-vitro data showing increased invasiveness of breast carcinoma cell lines (originally α6β4 negative) following transfection with full-length β4[24]. The β4 subunit introduced into these cells preferentially combines with the α6 subunit of endogenous α6β1, resulting in overexpression of α6β4[24].

Tumor cell invasion involves the formation of actin-containing motility structures such as lamellipodia and filopodia. It has been shown that EGF stimulation produces a redistribution of α6β4 integrin from hemidesmosomes to the lamellipodia and filopodia of invasive tumor cells[12,25-28]. The formation of these structures is dependent on PI3K[12,25,27]. Factors regulating the transition from adherent cells to invasive motile cells are poorly understood, but α6β4-mediated activation of the Ras-MAP kinase pathway may be important, as subsequent activation of myosin light chain kinase[29] leads to increased ATPase activity and contractility, which are fundamental to locomotion.

Multiple studies have shown significant crosstalk between α6β4 integrin and EGFR in carcinoma cells [12-14]. Following stimulation with EGF, the β4 integrin subunit becomes tyrosine phosphorylated [14,30], and α6β4 is mobilized from hemidesmosomes to actin-rich protrusions at the leading edge of motile cells[12]. At the leading edge, α6β4 signals through Rho to promote tumor cell migration, perhaps in part by activating Rho to stimulate acto-myosin contraction, necessary for generating traction in migrating cells[12,25,27]. EGFR has been shown to co-immunoprecipitate with α6β4[13], and EGFR is co-expressed with α6β4 in breast cancers that tend to metastasize to the lungs[11,31].

In a recent study, Lu et al. found that a 65-gene "β4 signature" derived from the top 0.1% of genes that correlated with β4 integrin subunit gene expression was associated with increased tumor recurrence and decreased patient survival when applied to four independent data sets [32]. The investigators hypothesized that a group of genes involved in α6β4 signaling was more likely to be associated with an adverse clinical outcome than α6β4 expression alone. In their study, EGFR was one of the top 10 genes associated with β4 integrin subunit gene expression.

Both α6β4 and EGFR are overexpressed in the basal subtype of breast cancers[11]. Recognized histologic variants of this basal subtype have a particular tendency to produce pulmonary metastases and cause early death [33-36]. MDA-MB-231 breast carcinoma cells express α6β4 and EGFR and have been shown to produce pulmonary metastases in nude mice[37]. The mechanism of α6β4-mediated pulmonary metastasis appears to involve recognition of hCLCA2, a β4-binding protein expressed in lung endothelial cells[38] that appears to serve as a specific vascular address for circulating tumor cells(12). If α6β4 functions, in part, to recognize this vascular address, EGFR may help to mediate the translocation of tumor cells into the adjacent tissue, as EGF has been shown to be a potent chemotactic factor for breast carcinoma cells [39,40].

We previously observed that antibody-mediated crosslinking of α6β4 in suspended MDA-MB-231 cells was sufficient to induce cell surface α6β4 clustering[20]. Crosslinking antibodies provide greater specificity than most integrin ligands[5], which typically interact with multiple different receptors. Clustering was significantly blocked when integrin crosslinking was performed in the presence of PI3K inhibitors, indicating that the clustering occurred through a PI3K-dependent mechanism[20]. In this report, we demonstrate that α6β4 crosslinking in nonadherent cells results in cell surface clustering of EGFR, selectively augmenting EGFR-mediated activation of Rho in response to EGF. As α6β4 signaling through Rho promotes tumor cell motility, a selective augmentation of EGFR-mediated Rho activation might further promote tumor cell migration. It is interesting that, although growth factor receptor signaling generally requires substrate adherence, the augmented response to EGF that we observed after crosslinking α6β4 and inducing EGFR clustering was observed in nonadherent cells. Augmented EGF signaling to Rho mediated by clustered EGFR may have relevance to chemotaxis and directed motility of nonadherent (circulating) or less adherent (migrating) tumor cells.

**Figure 5:**
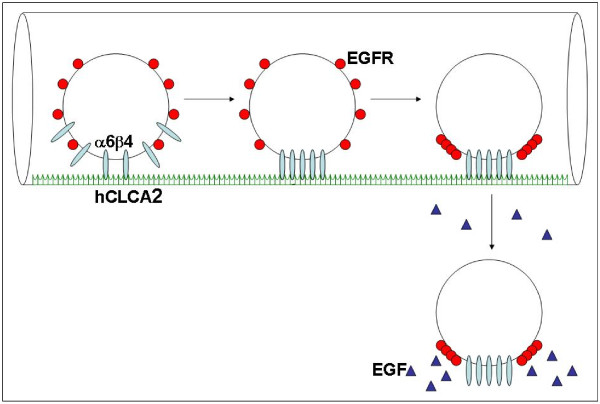
**Schematic diagram illustrating hypothetical role of integrin-induced EGFR clustering in tumor progression**. Circulating tumor cells might bind endothelial hCLCA2, crosslinking α6β4 and inducing EGFR clustering. Integrin-induced EGFR clustering enhances EGF-mediated activation of Rho, which is known to be involved in processes leading to cell motility and invasion. Clustered EGFR might favor directed motility towards EGF in the adjacent tissue.

We hypothesize that α6β4 integrin clustering at the leading edge of a tumor might lead to a redistribution and concentration of EGFR at the invading front, thereby promoting the motility of tumor cells towards an EGF gradient. Laminin-5, a principal matrix ligand for α6β4 integrin, is secreted and deposited in the connective tissues surrounding invasive carcinomas, facilitating the crosslinking of α6β4 at the invading front[41]. Alternatively, circulating tumor cells might bind endothelial hCLCA2, crosslinking α6β4 and inducing EGFR clustering. After homing to the lung vasculature, therefore, tumor cells with EGFR clustering might undergo an augmented response to EGF, favoring directed motility towards EGF in the adjacent lung tissue (Figure 5).

## Conclusion

Crosslinking α6β4 integrin in breast carcinoma cells induces EGFR clustering and preferentially promotes Rho activation in response to EGF, with only minimal effects on Akt and Erk 1,2 phosphorylation. This integrin-mediated selective augmentation of EGFR signaling might promote tumor cell cytoskeletal rearrangements important for tumor progression.

## Competing interests

PJM is Vice-President of Biology Applications at Amnis Corporation and owns stock in Amnis Corporation.

## Authors' contributions

MZG participated in the design of the study and supervised the fluorescence miscoscopy work, Western blot studies, and pull-down assays. XZ carried out the fluorescence microscopy and Western blot studies and prepared cells for the multispectral imaging studies. XL performed Western blot and Rho pull-down assays. PJM participated in the design of the multispectral imaging studies, BEH did technical work for the multispectral imaging studies, and both PJM and BEH helped to analyze the data. WAW helped with the interpretation of data and critical revision of the manuscript. All authors read and approved the final manuscript.

## References

[B1] Hynes RO (2002). Integrins: bidirectional, allosteric signaling machines. Cell.

[B2] Miranti CK, Brugge JS (2002). Sensing the environment: a historical perspective on integrin signal transduction. Nat Cell Biol.

[B3] Hughes PE, Pfaff M (1998). Integrin affinity modulation. Trends Cell Biol.

[B4] Ridley AJ, Schwartz MA, Burridge K, Firtel RA, Ginsberg MH, Borisy G, Parsons JT, Horwitz AR (2003). Cell migration: integrating signals from front to back. Science.

[B5] Miyamoto S, Akiyama SK, Yamada KM (1995). Synergistic roles for receptor occupancy and aggregation in integrin transmembrane function. Science.

[B6] Stewart MP, McDowall A, Hogg N (1998). LFA-1-mediated adhesion is regulated by cytoskeletal restraint and by a Ca2+-dependent protease, calpain. J Cell Biol.

[B7] van Kooyk Y, van Vliet SJ, Figdor CG (1999). The actin cytoskeleton regulates LFA-1 ligand binding through avidity rather than affinity changes. J Biol Chem.

[B8] Schwartz MA, Ginsberg MH (2002). Networks and crosstalk: integrin signalling spreads. Nat Cell Biol.

[B9] Miyamoto S, Teramoto H, Gutkind JS, Yamada KM (1996). Integrins can collaborate with growth factors for phosphorylation of receptor tyrosine kinases and MAP kinase activation: roles of integrin aggregation and occupancy of receptors. J Cell Biol.

[B10] Shaw LM (1999). Integrin function in breast carcinoma progression. J Mammary Gland Biol Neoplasia.

[B11] Perou CM, Sorlie T, Eisen MB, Rijn M van de, Jeffrey SS, Rees CA, Pollack JR, Ross DT, Johnsen H, Akslen LA, Fluge O, Pergamenschikov A, Williams C, Zhu SX, Lønning PE, Børresen-Dale AL, Brown PO, Botstein D (2000). Molecular portraits of human breast tumours. Nature.

[B12] Rabinovitz I, Toker A, Mercurio AM (1999). Protein kinase C-dependent mobilization of the alpha6beta4 integrin from hemidesmosomes and its association with actin-rich cell protrusions drive the chemotactic migration of carcinoma cells. J Cell Biol.

[B13] Mariotti A, Kedeshian PA, Dans M, Curatola AM, Gagnoux-Palacios L, Giancotti FG (2001). EGF-R signaling through Fyn kinase disrupts the function of integrin alpha6beta4 at hemidesmosomes: role in epithelial cell migration and carcinoma invasion. J Cell Biol.

[B14] Mainiero F, Pepe A, Yeon M, Ren Y, Giancotti FG (1996). The intracellular functions of alpha6beta4 integrin are regulated by EGF. J Cell Biol.

[B15] Peterson EJ, Woods ML, Dmowski SA, Derimanov G, Jordan MS, Wu JN, Myung PS, Liu QH, Pribila JT, Freedman BD, Shimizu Y, Koretzky GA (2001). Coupling of the TCR to integrin activation by Slap-130/Fyb. Science.

[B16] Ferguson KM (2004). Active and inactive conformations of the epidermal growth factor receptor. Biochem Soc Trans.

[B17] Ichinose J, Murata M, Yanagida T, Sako Y (2004). EGF signalling amplification induced by dynamic clustering of EGFR. Biochem Biophys Res Commun.

[B18] Bray D, Levin MD, Morton-Firth CJ (1998). Receptor clustering as a cellular mechanism to control sensitivity. Nature.

[B19] Crouch MF, Davy DA, Willard FS, Berven LA (2000). Insulin induces epidermal growth factor (EGF) receptor clustering and potentiates EGF-stimulated DNA synthesis in swiss 3T3 cells: a mechanism for costimulation in mitogenic synergy. Immunol Cell Biol.

[B20] Gilcrease MZ, Zhou X, Welch K (2004). Adhesion-independent alpha6beta4 integrin clustering is mediated by phosphatidylinositol 3-kinase. Cancer Res.

[B21] Hogervorst F, Kuikman I, van Kessel AG, Sonnenberg A (1991). Molecular cloning of the human alpha 6 integrin subunit. Alternative splicing of alpha 6 mRNA and chromosomal localization of the alpha 6 and beta 4 genes. Eur J Biochem.

[B22] Dowling J, Yu QC, Fuchs E (1996). Beta4 integrin is required for hemidesmosome formation, cell adhesion and cell survival. J Cell Biol.

[B23] Nagato T, Yoshida H, Yoshida A, Uehara Y (1980). A scanning electron microscope study of myoepithelial cells in exocrine glands. Cell Tissue Res.

[B24] Shaw LM, Rabinovitz I, Wang HH, Toker A, Mercurio AM (1997). Activation of phosphoinositide 3-OH kinase by the alpha6beta4 integrin promotes carcinoma invasion. Cell.

[B25] O'Connor KL, Nguyen BK, Mercurio AM (2000). RhoA function in lamellae formation and migration is regulated by the alpha6beta4 integrin and cAMP metabolism. J Cell Biol.

[B26] Rabinovitz I, Mercurio AM (1997). The integrin alpha6beta4 functions in carcinoma cell migration on laminin-1 by mediating the formation and stabilization of actin-containing motility structures. J Cell Biol.

[B27] Rabinovitz I, Gipson IK, Mercurio AM (2001). Traction forces mediated by alpha6beta4 integrin: implications for basement membrane organization and tumor invasion. Mol Biol Cell.

[B28] O'Connor KL, Shaw LM, Mercurio AM (1998). Release of cAMP gating by the alpha6beta4 integrin stimulates lamellae formation and the chemotactic migration of invasive carcinoma cells. J Cell Biol.

[B29] Mainiero F, Murgia C, Wary KK, Curatola AM, Pepe A, Blumemberg M, Westwick JK, Der CJ, Giancotti FG (1997). The coupling of alpha6beta4 integrin to Ras-MAP kinase pathways mediated by Shc controls keratinocyte proliferation. Embo J.

[B30] Russell AJ, Fincher EF, Millman L, Smith R, Vela V, Waterman EA, Dey CN, Guide S, Weaver VM, Marinkovich MP (2003). Alpha 6 beta 4 integrin regulates keratinocyte chemotaxis through differential GTPase activation and antagonism of alpha 3 beta 1 integrin. J Cell Sci.

[B31] van 't Veer LJ, Dai H, Vijver MJ van de, He YD, Hart AA, Mao M, Peterse HL, Kooy K van der, Marton MJ, Witteveen AT, Schreiber GJ, Kerkhoven RM, Roberts C, Linsley PS, Bernards R, Friend SH (2002). Gene expression profiling predicts clinical outcome of breast cancer. Nature.

[B32] Lu S, Simin K, Khan A, Mercurio AM (2008). Analysis of Integrin {beta}4 Expression in Human Breast Cancer: Association with Basal-like Tumors and Prognostic Significance. Clin Cancer Res.

[B33] Leibl S, Gogg-Kammerer M, Sommersacher A, Denk H, Moinfar F (2005). Metaplastic breast carcinomas: are they of myoepithelial differentiation?: immunohistochemical profile of the sarcomatoid subtype using novel myoepithelial markers. Am J Surg Pathol.

[B34] Wargotz ES, Norris HJ (1989). Metaplastic carcinomas of the breast. III. Carcinosarcoma. Cancer.

[B35] Tsuda H, Takarabe T, Hasegawa F, Fukutomi T, Hirohashi S (2000). Large, central acellular zones indicating myoepithelial tumor differentiation in high-grade invasive ductal carcinomas as markers of predisposition to lung and brain metastases. Am J Surg Pathol.

[B36] Jimenez RE, Wallis T, Visscher DW (2001). Centrally necrotizing carcinomas of the breast: a distinct histologic subtype with aggressive clinical behavior. Am J Surg Pathol.

[B37] Mukhopadhyay R, Theriault RL, Price JE (1999). Increased levels of alpha6 integrins are associated with the metastatic phenotype of human breast cancer cells. Clin Exp Metastasis.

[B38] Abdel-Ghany M, Cheng HC, Elble RC, Pauli BU (2001). The breast cancer beta 4 integrin and endothelial human CLCA2 mediate lung metastasis. J Biol Chem.

[B39] Price JT, Tiganis T, Agarwal A, Djakiew D, Thompson EW (1999). Epidermal growth factor promotes MDA-MB-231 breast cancer cell migration through a phosphatidylinositol 3'-kinase and phospholipase C-dependent mechanism. Cancer Res.

[B40] Yang Z, Bagheri-Yarmand R, Wang RA, Adam L, Papadimitrakopoulou VV, Clayman GL, El-Naggar A, Lotan R, Barnes CJ, Hong WK, Kumar R (2004). The epidermal growth factor receptor tyrosine kinase inhibitor ZD1839 (Iressa) suppresses c-Src and Pak1 pathways and invasiveness of human cancer cells. Clin Cancer Res.

[B41] Giannelli G, Antonaci S (2000). Biological and clinical relevance of Laminin-5 in cancer. Clin Exp Metastasis.

